# Markers of Negative Emotionality in Individuals With Comorbid Alcohol Use Disorder and Post‐Traumatic Stress Disorder: Role of Childhood Trauma

**DOI:** 10.1111/adb.70037

**Published:** 2025-04-18

**Authors:** E. C. Cullins, T. Gunawan, M. L. Schwandt, J. W. Luk, D. T. George, N. Diazgranados, D. Goldman, V. A. Ramchandani

**Affiliations:** ^1^ Human Psychopharmacology Laboratory, National Institute on Alcohol Abuse and Alcoholism, National Institutes of Health Bethesda Maryland USA; ^2^ Department of Neuroscience The University of Texas at Austin Austin Texas USA; ^3^ Office of the Clinical Director, National Institute on Alcohol Abuse and Alcoholism National Institutes of Health Bethesda Maryland USA; ^4^ Laboratory of Neurogenetics, National Institute on Alcohol Abuse and Alcoholism National Institutes of Health Bethesda Maryland USA

**Keywords:** alcohol use, alcohol use disorder, allostatic load, childhood trauma, negative emotionality, post‐traumatic stress disorder

## Abstract

Post‐traumatic stress disorder (PTSD) and alcohol use disorder (AUD) are characterized with heightened negative emotionality (NE) and are frequently comorbid. However, little research has investigated NE in individuals with comorbid AUD/PTSD. We compared psychological and biological markers of NE phenotypes, and alcohol‐related outcomes between individuals with AUD with and without PTSD, and healthy controls. Additionally, we evaluated whether childhood trauma severity moderated these relationships. Participants [*N* = 1292; healthy controls (HC): *n* = 502 (38.9%); AUD only: *n* = 610 (47.2%), and AUD/PTSD (CMB); *n* = 180 (13.9%)] enrolled in the National Institute on Alcohol Abuse and Alcoholism Natural History Protocol underwent clinical, biological and behavioural phenotyping that included psychiatric diagnoses, markers of negative emotionality and allostatic load, alcohol use behaviour, and history of childhood trauma. The CMB group had the most severe alcohol use and childhood trauma history. Psychological NE were the most dysregulated among the CMB group. Biological markers of NE were also dysregulated among the AUD and CMB group, where they displayed greater resting heart rate, diastolic blood pressure and HDL cholesterol relative to HC. Greater childhood trauma severity was associated with greater psychological NE. However, the childhood trauma did not moderate any relationship between diagnosis and NE phenotypes. These results highlight important differences in NE, childhood trauma and alcohol use in individuals with AUD with and without comorbid PTSD. Targeting NE and alcohol‐related behaviours is critical in effective treatment of individuals with comorbid AUD/PTSD.

**Trial Registration:** ClinicalTrials.gov: NCT02231840.

## Graphical abstract

Individuals with comorbid alcohol use disorder (AUD) and post‐traumatic stress disorder (PTSD) exhibit dysregulated psychological and biological markers of negative emotionality (NE), and more severe alcohol use behaviors than those with AUD alone. Childhood trauma was associated with greater NE but did not amplify differences between diagnostic groups. These findings underscore the importance of addressing NE and trauma in treatments for comorbid AUD/PTSD.

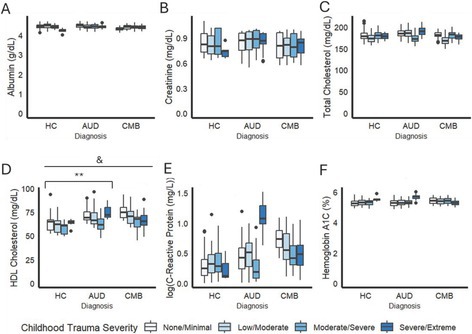

## 1 Introduction

Post‐traumatic stress disorder (PTSD) and alcohol use disorder (AUD) are often comorbid. Individuals with PTSD are at a three‐fold risk of developing AUD relative to those without PTSD [1]. Individuals with comorbid AUD and PTSD exhibit significantly poorer social and psychiatric functioning, worse treatment outcomes, and higher relapse rates than those with either individual disorder [2, 3, 4]. Given the substantial public health burden associated with this comorbidity, there is a critical need for more effective and targeted treatments for this population.

A key shared feature of PTSD and AUD is negative emotionality (NE), which includes elevated stress reactivity, anxiety and negative mood states. Cross‐diagnostic frameworks such as the Addictions Neuroclinical Assessment (ANA) highlights NE as a transdiagnostic risk factor for addiction and related psychopathology [5, 6]. While there is evidence of heightened NE in individuals with PTSD and AUD separately [7], few studies have investigated NE phenotypes in the comorbid AUD/PTSD population [8].

To investigate the full spectrum of the NE phenotype in individuals with AUD/PTSD, we sought to include both psychological and biological markers of NE. Dysregulation of the brain's stress response system is a core feature underlying NE [9]. Changes within these systems can be indicated through measures of allostatic load, which quantifies the burden of accumulated stress on the body and is positively associated with NE [10]. Therefore, we evaluated psychological measures of NE (depression, anxiety, perceived stress) and biomarkers of allostatic load [11] [heart rate (HR), blood pressure (BP), total and high‐density lipoprotein (HDL) cholesterol, haemoglobin A1C (Hgb‐A1C), C‐reactive protein (CRP), albumin and creatinine] across diagnostic groups.

In addition to NE, childhood trauma is a critical risk factor for both AUD and PTSD [12, 13]. Childhood trauma sensitizes NE systems, increasing susceptibility to both disorders and is associated with increased NE and allostatic load in adulthood [14, 15, 16]. However, it remains unclear whether childhood trauma severity exacerbates NE dysregulation in individuals with comorbid AUD/PTSD.

Our primary objective for this study was to compare NE and alcohol‐related outcomes between individuals with AUD, comorbid AUD/PTSD and individuals without either disorder. We hypothesized that AUD/PTSD group would exhibit greater severity of psychological and biological markers of NE, and more severe alcohol use patterns relative to AUD‐only and healthy control (HC) groups. Our secondary objective was to assess whether childhood trauma severity moderated the differences in NE and alcohol‐related outcomes between groups. We hypothesized that individuals with greater childhood trauma severity would exhibit greater NE and more severe alcohol use patterns, and that childhood trauma severity would exacerbate the differences between diagnostic groups.

## 2 Materials and Methods

### 2.1 Participants

Participants (*N* = 1292) completed the National Institute on Alcohol Abuse and Alcoholism (NIAAA) Natural History Protocol between January 2015 to October 2022 at the National Institutes of Health (NIH) Clinical Center in Bethesda, MD. Four hundred thirty‐nine individuals (34.0%) were seeking treatment for AUD and were admitted into the inpatient alcohol treatment unit at the NIH Clinical Center. The sample was stratified into three diagnostic groups based on their lifetime AUD/PTSD comorbidity: healthy controls [HC; *n* = 502 (38.9%)], AUD without PTSD [AUD; *n* = 610 (47.2%)] and AUD with comorbid PTSD [CMB; *n* = 180 (13.9%)]. The PTSD only group was not included in the analyses due to the small group size in the present sample (*n* = 15). The protocol was approved by the Institutional Review Board at the NIH. Informed consent was obtained from all participants.

### 2.2 Measures

#### 2.2.1 Psychiatric Diagnoses

Psychiatric diagnoses were determined using the Structured Clinical Interview for DSM‐IV (SCID‐IV; [17]) or DSM‐5 (SCID‐5; [18]). *N* = 409 individuals underwent the SCID‐IV, while *N* = 883 individuals underwent the SCID‐5, after it was implemented in the protocol in 2017. Individuals were diagnosed with PTSD if they met the DSM‐IV or DSM‐5 criteria for PTSD. Individuals who met the DSM‐IV criteria for alcohol abuse or alcohol dependence, or met the DSM‐5 criteria for AUD, were considered to have AUD.

#### 2.2.2 Childhood Trauma

Childhood traumatic stress was assessed using the Childhood Trauma Questionnaire (CTQ; [19]). There are five subscales in the CTQ:emotional abuse, physical abuse, sexual abuse, emotional neglect and physical neglect. Severity level was grouped into four categories based on total scores: none/minimal (≤ 36), low/moderate (> 36 and ≤ 51), moderate/severe (> 51 and ≤ 68) and severe/extreme (≥ 69) [20].

#### 2.2.3 Psychological NE Phenotypes

Psychological phenotypes of NE include perceived stress, trait anxiety and depression. Perceived stress was evaluated using the Perceived Stress Scale (PSS), a 10‐question inventory that measures how often an individual feels stressful in different situations in the last month [21]. Trait anxiety was assessed with the State–Trait Anxiety Inventory, a 40‐item inventory that measures state and trait anxiety in adults [19]. Depression was measured using the Montgomery‐Åsberg Depression Rating Scale (MADRS), a 10‐item inventory of common depressive symptoms [22].

#### 2.2.4 Physiological and Biological NE Phenotypes

To assess physiological and biological phenotypes of NE, we measured resting heart rate, blood pressure and biomarkers of allostatic load. Resting heart rate and systolic and diastolic blood pressure were obtained upon admission to the inpatient unit for treatment‐seeking patients and upon arrival to the NIAAA clinic for nontreatment seeking participants. Blood samples to assess biomarkers of allostatic load were obtained on the morning after admission to the inpatient unit for treatment‐seeking patients and upon arrival to the NIAAA clinic for nontreatment seeking participants. Biomarkers of allostatic load include serum albumin, creatinine, total and HDL cholesterol, Hgb‐A1C and CRP [11].

#### 2.2.5 Alcohol use Outcomes

Problematic drinking was assessed with the Alcohol Use Disorders Identification Test (AUDIT; [23]). Alcohol use disorder severity was assessed with the Alcohol Dependence Scale (ADS; [24]). The Obsessive‐Compulsive Drinking Scale (OCDS) was used to measure obsessive and compulsive drinking behaviours [25]. Number of heavy drinking days in the past 90 days was evaluated using the timeline follow‐back (TLFB) method [26]. Finally, age of first alcohol use and total lifetime drinks were obtained using the Lifetime Drinking History questionnaire (LDH; [27]).

### 2.3 Statistical Analysis

All analyses were performed using R statistical package (Version 4.2.1). Analysis of variance (ANOVAs) and chi‐square tests were used to compare sociodemographic characteristics between diagnostic groups for continuous and categorical variables, respectively. Analysis of covariance (ANCOVA) was used to test for differences between diagnostic groups (HC vs. AUD vs. CMB) and to test the moderating effect of childhood trauma severity on outcomes, with age, gender, race and alcohol withdrawal [Clinical Institute Withdrawal Assessment for Alcohol Withdrawal (CIWA‐Ar)] scores as covariates. CIWA‐Ar scores were not included as a covariate for alcohol outcomes. Significant main and interaction effects were followed up with post‐hoc tests. Missing data rates were low (≤ 10%). Little's MCAR test [28] indicated that missing data rates for the PSS (8% missing) and ADS (7% missing) were different between diagnostic groups. We used multiple imputation using pooled estimates from 20 imputed datasets to impute missing data. The Benjamini–Hochberg procedure [29] was applied to control for false discovery rate. For all analyses, a cutoff of *p* < 0.05 was used to indicate statistical significance.

## 3 Results

### 3.1 Demographics

Table 1 includes participant demographics and characteristics by diagnostic group. Participants had a mean age of 40.6 years (SD = 13.5), with 41.6% (*n* = 537) identifying as female. Participants identified as African American (41.6%), Asian (5.7%), American Indian/Alaskan Native (0.7%), Multiracial (3.3%), Native Hawaiian/Pacific Islander (< 0.1%), Unknown (4.6%) and White/European American (44.0%) Due to small percentages of individuals in racial minority categories, we divided our sample into three racial categories for analyses: White/European American, Black/African American and Other. Mean number of years of education were 14.8 (SD = 3.3). Household income was divided into three brackets: < $20 000 per year (26.1%; *n* = 337), $20 000 to $75 000 per year (43.9%; *n* = 567) and > $75 000 per year (26.5%; *n* = 342).

**TABLE 1:** Sample characteristics by diagnosis.

Demographics	HC, *n* = 502 (38.9%)	AUD, *n* = 610 (47.2%)	CMB, *n* = 180 (13.9%)
Age in years, mean (± SD)	36.9 (± 13.9)	42.5 (± 13.1)	44.5 (± 11.3)
Female, *n* (%)	271 (54.0)	185 (30.3)	81 (45.0)
Male, *n* (%)	231 (46.0)	425 (69.7)	99 (55.0)
Asian, *n* (%)	49 (9.8)	23 (3.8)	2 (1.1)
American Indian or Alaska Native, *n* (%)	2 (0.4)	3 (0.5)	4 (2.2)
Black/African American, *n* (%)	196 (39.0)	262 (43.0)	79 (43.9)
Multiracial, *n* (%)	14 (2.8)	22 (3.6)	7 (3.9)
Native Hawaiian or other Pacific Islander, *n* (%)	1 (0.2)	0 (0.0)	0 (0.0)
Unknown, *n* (%)	18 (3.6)	30 (4.9)	12 (6.7)
White/European American, *n* (%)	222 (44.2)	270 (44.3)	76 (42.2)
Hispanic, *n* (%)	44 (8.8)	53 (8.7)	14 (7.8)
Years of education, mean (± SD)	16.1 (± 3.2)	14.2 (± 3.2)	13.4 (± 2.6)
Household income ($/year), *n* (%)			
< 20 000	65 (12.9)	196 (32.1)	76 (42.2)
20 000–75 000	246 (49.0)	263 (43.1)	58 (32.2)
> 75 000	180 (35.9)	128 (20.9)	34 (18.9)
Treatment‐seeking for AUD, *n* (%)	0	304 (49.8)	134 (74.4)
CIWA‐Ar score, mean (± SD)	0.7 (2.4)	2.2 (3.6)	3.6 (4.4)
Childhood trauma severity, *n* (%)			
None/minimal	363 (72.3)	329 (53.9)	33 (18.3)
Low/moderate	92 (18.3)	155 (25.4)	44 (24.4)
Moderate/severe	25 (5.0)	55 (9.0)	41 (22.8)
Severe/extreme	6 (1.2)	23 (3.8)	41 (22.8)

Abbreviations: AUD, alcohol use disorder; CIWA‐Ar, Clinical Institute Withdrawal Assessment of Alcohol Scale, Revised; CMB, comorbid AUD/PTSD; HC, healthy control; SD, standard deviation.

Individuals in the CMB and AUD groups were significantly older than those in the HC group (*p*s < 0.001). There was a lower percentage of females in the AUD group relative to the HC group (30.3% vs. 54.0%), while the CMB group had relatively equal percentages of males and females (45.0% female). There were no significant differences in the racial distributions between the diagnostic groups. Individuals in the CMB group had significantly fewer years of education than those in the AUD (*p* < 0.01) and HC groups (*p* < 0.001), while individuals in the AUD group had significantly less years of education than the HC group (*p* < 0.001). Those in the CMB group were more likely to be in the lowest income bracket (42.2%) relative to AUD (32.1%) and HC (12.9%) groups. Individuals in the CMB group had a significantly higher percentage of individuals seeking treatment for AUD (74.4%) than the AUD only group (49.8%). CIWA scores were highest among the CMB group compared with the AUD and HC groups (*p*s < 0.001). The AUD group had higher CIWA scores than the HC group (*p* < 0.001).

**FIGURE 1:**
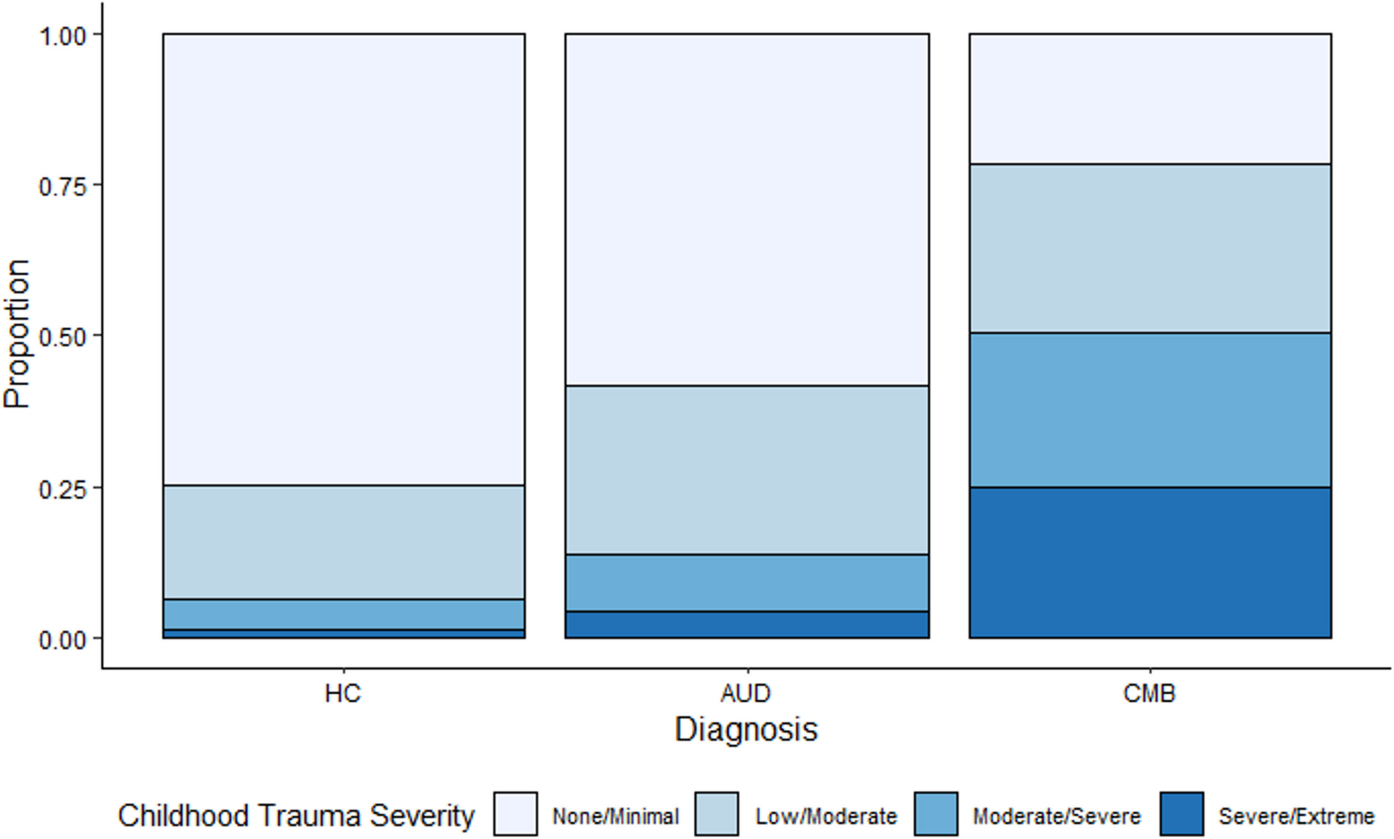
Proportion of individuals in each childhood trauma severity by diagnostic groups.

Childhood trauma severity significantly differed between diagnostic groups (*χ* ^2^(6) = 232.08, *p* < 0.001). Individuals in the CMB group were more likely to endorse severe/extreme childhood trauma, while those in the HC group were more likely to endorse none/minimal childhood trauma. Individuals in the AUD group had relatively equal endorsement for each severity categories (Table 1, Figure 1). These results were consistent across all trauma subscales physical abuse: *χ* ^2^(6) = 127.03, emotional abuse: *χ* ^2^(6) = 185.36, sexual abuse: *χ* ^2^(6) = 155.26, physical neglect: *χ* ^2^(6) = 104.88, emotional neglect: *χ* ^2^(6) = 104.13; *p*s < 0.001.

### 3.2 Psychological NE Phenotypes

**FIGURE 2:**
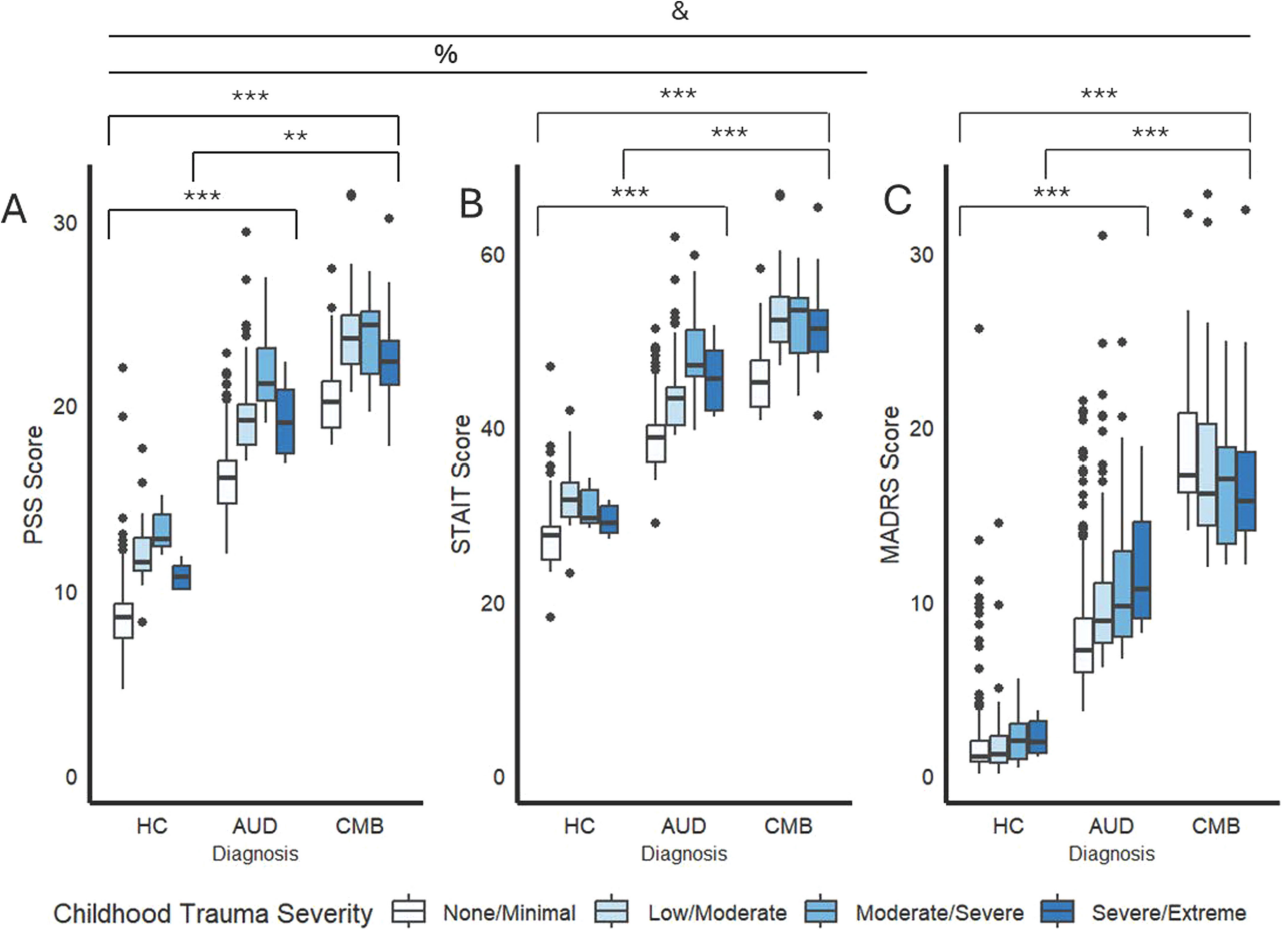
Associations of diagnosis and childhood trauma severity with perceived stress (A), depression (B) and trait anxiety (C). Statistics performed by ANCOVA with age, gender, race and alcohol withdrawal as covariates. ‘&’ indicates a main effect of diagnosis at *p* < 0.05, ‘%’ indicates a main effect of childhood trauma severity at *p* < 0.05. ***Significant pair‐wise comparison at *p* < 0.001.

Psychological NE phenotypes significantly differed between diagnostic groups (depression: *F*(2, 1275) = 90.54, *p* < 0.001, η^2^ = 0.11; anxiety: *F*(2, 1275) = 104.18, *p* < 0.001, η^2^ = 0.13; perceived stress: *F*(2, 1275) = 98.78, *p* < 0.001, η^2^ = 0.12; Figure 2, Table S1). All psychological NE phenotypes were greater in individuals in the CMB group than those in the AUD and HC groups (*p*s < 0.001), and individuals in the AUD group displayed higher levels of psychological NE phenotypes than the HC group (*p*s < 0.001). Anxiety and perceived stress, but not depression were also linearly associated with childhood trauma severity (depression: *F*(3, 1275) = 0.61, *p* = 0.61; anxiety: *F*(3, 1275) = 8.10, *p* < 0.001, η^2^ = 0.01; perceived stress: *F*(3, 1275) = 11.51, *p* < 0.001, η^2^ = 0.02), whereby greater childhood trauma severity was associated with greater levels of anxiety and perceived stress (Figure 2). Childhood trauma severity did not moderate the relationship between diagnosis and any psychological outcomes (all *p*s > 0.05).

### 3.3 Physiological and Biological NE Phenotypes

**FIGURE 3:**
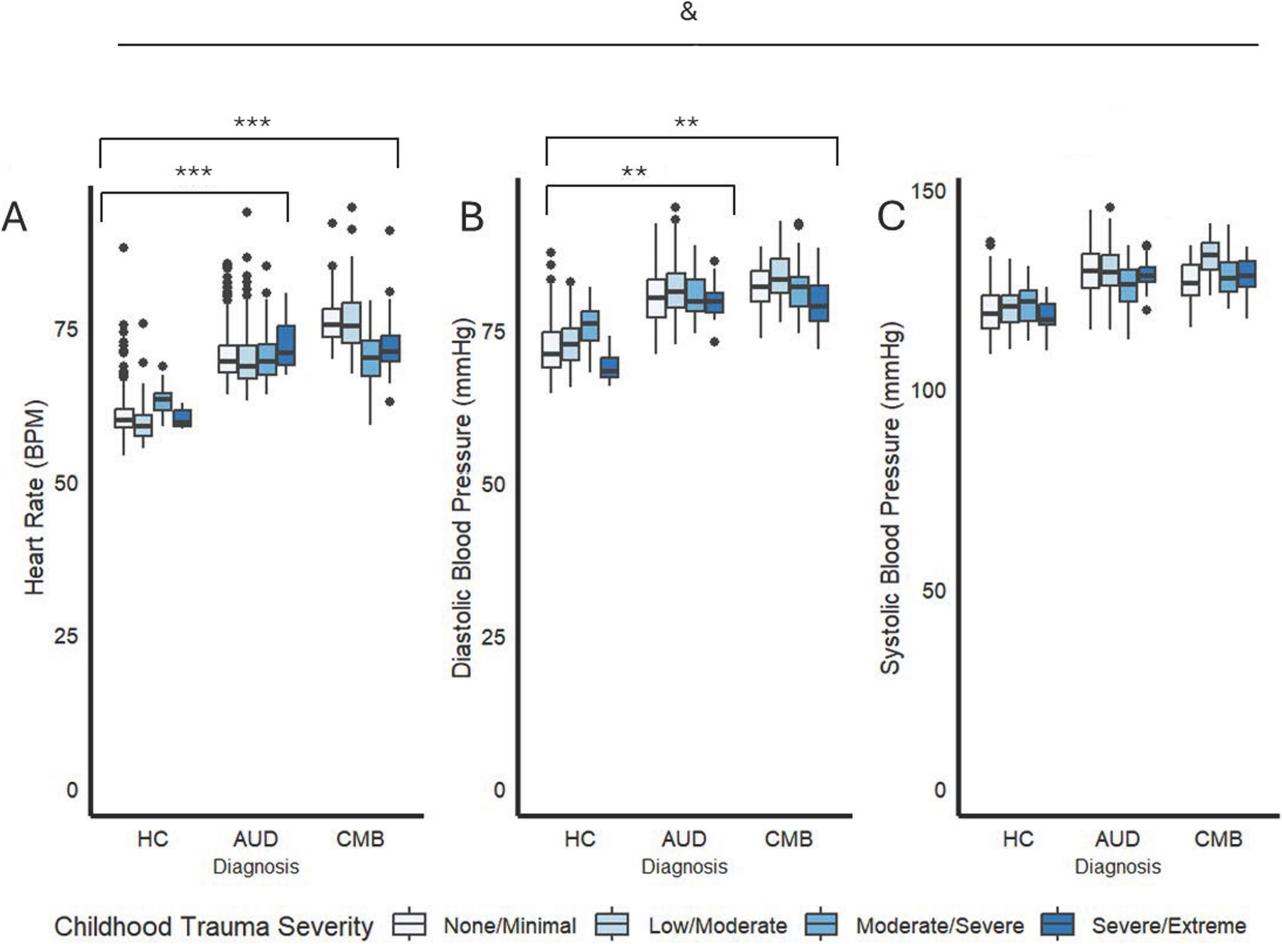
Associations of diagnosis and childhood trauma severity with resting heart rate (A), diastolic blood pressure (B) and systolic blood pressure (C). Statistics performed by ANCOVA with age, gender, race, and alcohol withdrawal as covariates. ‘&’ indicates a main effect of diagnosis at *p* < 0.05. **Significant pair‐wise comparison at *p* < 0.01. ***Significant pair‐wise comparison at *p* < 0.001.

Physiological NE phenotypes differed between diagnostic groups (resting HR: *F*(2, 1275) = 37.27, *p* < 0.001, η^2^ = 0.05; diastolic BP: *F*(2, 1275) = 19.86, *p* < 0.001, η^2^ = 0.03; systolic BP: *F*(2, 1275) = 13.98, *p* < 0.001, η^2^ = 0.02). Resting HR were higher among individuals in the CMB and AUD groups than individuals in HC (*p*s < 0.001). Diastolic BP were also higher in individuals in the CMB and AUD groups than the HC group (*p*s = 0.002; Figure 3, Table S1). Differences in systolic BP did not survive multiple comparison correction (*p*s = 0.057). Childhood trauma severity did not moderate the relationship between diagnosis and physiological NE phenotypes (all *p*s > 0.05).

**FIGURE 4:**
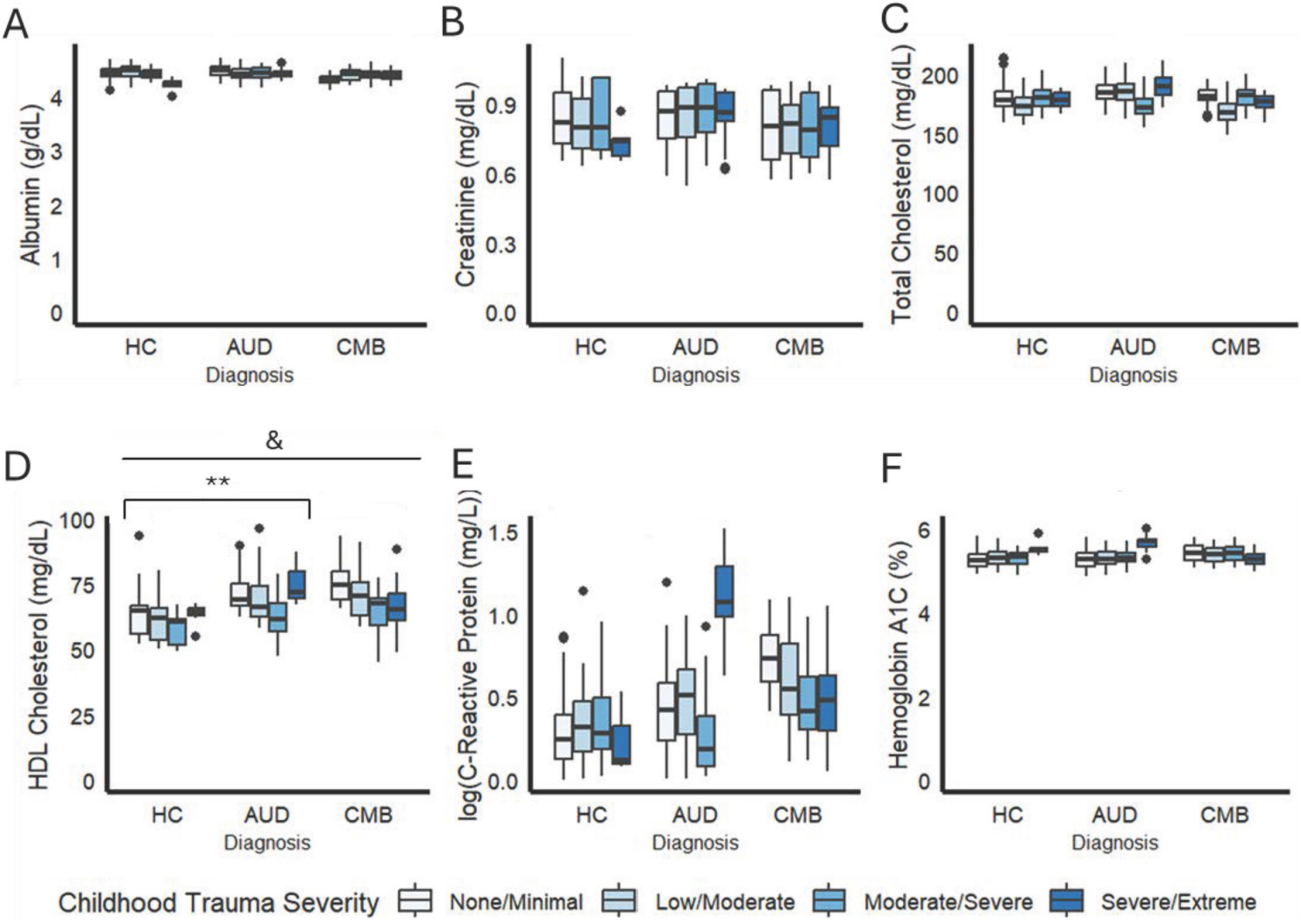
Associations of diagnosis and childhood trauma severity with albumin (A), creatinine (B), total cholesterol (C), HDL cholesterol (D), C‐reactive protein (E) and haemoglobin A1C (F). Statistics performed by ANCOVA with age, gender, race and alcohol withdrawal as covariates. ‘&’ indicates a main effect of diagnosis at *p* < 0.05. **Significant pair‐wise comparison at *p* < 0.01.

Creatinine, HDL cholesterol and CRP were the only NE biomarkers that showed a significant effect of diagnostic groups (albumin: *F*(2, 1275) = 2.97, *p* = 0.051, η^2^ = 0.004; creatinine: *F*(2, 1275) = 15.17, *p* < 0.001, η^2^ = 0.02; total cholesterol: *F*(2, 1275) = 0.42, *p* < 0.05, η^2^ = 0.0006; HDL cholesterol: *F*(2, 1275) = 14.59, *p* < 0.001, η^2^ = 0.02, CRP: *F*(2, 1275) = 5.63, *p* = 0.004, η^2^ = 0.008; Hgb‐A1C: *F*(2, 1275) = 1.69, *p* > 0.05. η^2^ = 0.002). HDL cholesterol was significantly higher among the AUD group compared to HC (*p* = 0.003). No other pairwise comparisons survived multiple comparison correction. A summary of biomarker levels across diagnostic groups can be found in Figure 4 and Table S1. Childhood trauma severity did not moderate the relationship between diagnosis and biological NE phenotypes.

### 3.4 Alcohol Outcomes

**FIGURE 5:**
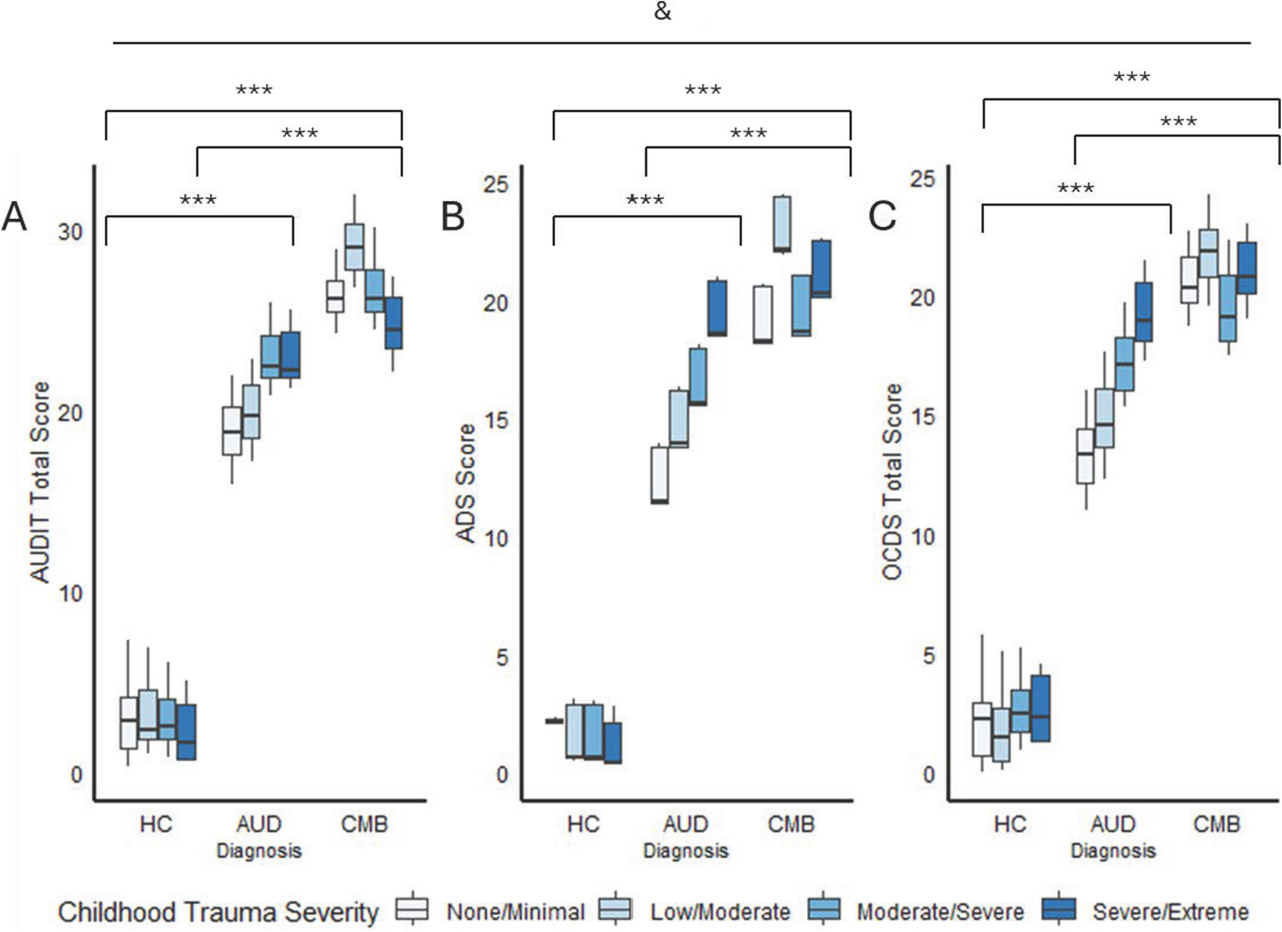
Associations of diagnosis and childhood trauma severity with AUDIT (A), ADS (B) and OCDS (C) total scores. Statistics performed by ANCOVA with age, gender, and race as covariates. & indicates a main effect of diagnosis at *p* < 0.05. *Significant pair‐wise comparison at *p* < 0.05. **Significant pair‐wise comparison at *p* < 0.01. ***Significant pair‐wise comparison at *p* < 0.001.

**FIGURE 6:**
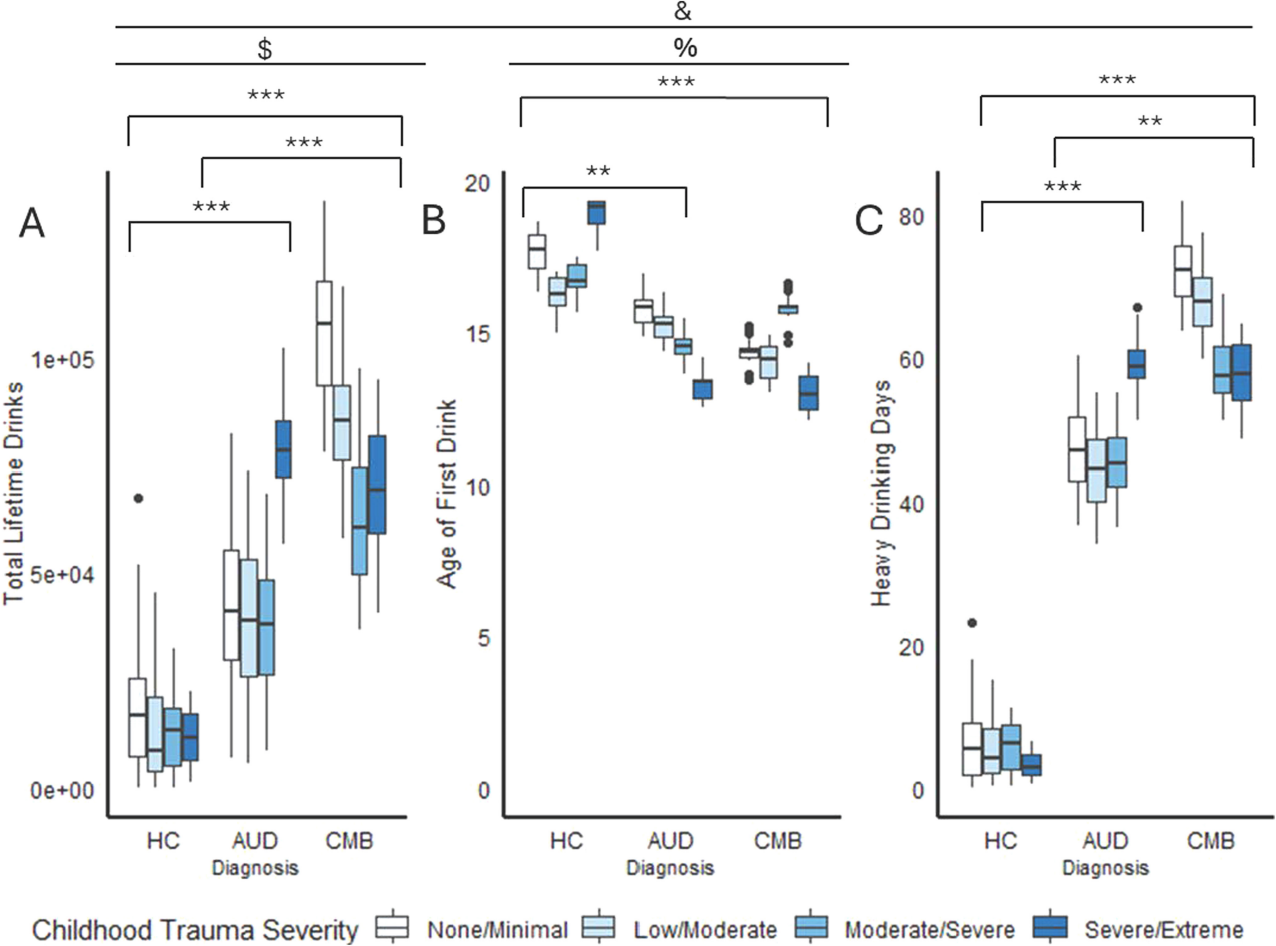
Associations of diagnosis and childhood trauma severity with total lifetime drinks (A), age of first drink (B) and heavy drinking days (C). Statistics performed by ANCOVA with age, gender and race as covariates. ‘&’ indicates a main effect of diagnosis at *p* < 0.05. ‘%’ indicates a main effect of childhood trauma severity at *p* < 0.05. ‘$’ indicates an interaction between diagnosis and childhood trauma severity at *p* < 0.05. *Significant pair‐wise comparison at *p* < 0.05. **Significant pair‐wise comparison at *p* < 0.01. ***Significant pair‐wise comparison at *p* < 0.001.

There were significant main effects of diagnosis for ADS, OCDS and AUDIT scores, as well as heavy drinking days, age of first drink and total lifetime drinks (ADS: *F*(2, 1275) = 176.70, *p* < 0.001, η^2^ = 0.19; OCDS: *F*(2, 1275) = 189.27, *p* < 0.001, η^2^ = 0.20; AUDIT: *F*(2, 1275) = 279.51, *p* < 0.001, η^2^ = 0.27; heavy drinking days: *F*(2, 1275) = 203.44, *p* < 0.001, η^2^ = 0.22; total lifetime drinks: *F*(2, 1275) = 63.10, *p* < 0.001, η^2^ = 0.08; age of first drink: *F*(2, 1275) = 13.87, *p* < 0.001, η^2^ = 0.02; Figure 5, Figure 6, Table S1). Individuals in the CMB group displayed greater ADS, OCDS and AUDIT scores, heavy drinking days and total lifetime drinks the AUD group (all *p*s < 0.01). The AUD group scored higher on all measures and lower on age of first drink than the HC group (all *p*s < 0.001). Main effects of childhood trauma severity were observed for only age of first drink (ADS: *F*(3, 1275) = 0.53, *p* > 0.05, η^2^ < 0.001; OCDS: *F*(3, 1275) = 0.36, *p* > 0.05, η^2^ < 0.001; AUDIT: *F*(3, 1275) = 0.24, *p* > 0.05, η^2^ < 0.001; heavy drinking days: *F*(2, 1275) = 0.03, *p* > 0.05, η^2^ < 0.001; total lifetime drinks: *F*(2, 1275) = 0.15, *p* > 0.05, η^2^ < 0.001; age of first drink: *F*(3, 1275) = 4.406.22, *p* = 0.004, η^2^ = 0.01). Those with greater childhood trauma severity exhibited lower age of first drink (all *p*s < 0.05). Significant interaction effects were observed for total lifetime drinks [*F*(6, 1275) = 3.95, *p* = 0.0006, η^2^ = 0.01], such that for individuals with comorbid AUD and PTSD, those with none/minimal childhood trauma reported greater total lifetime drinks than those with moderate/severe (*p* < 0.001) and severe/extreme childhood trauma (*p* = 0.009).

## 4 Discussion

This study aimed to assess differences in psychological, physiological and biological NE phenotypes between individuals with AUD and without PTSD, individuals with comorbid AUD/PTSD and individuals without either disorder. Additionally, we evaluated the moderating role of childhood trauma severity between diagnosis and NE phenotypes. Consistent with our first hypothesis, heightened NE were detected at the psychological, physiological and biological levels among individuals with comorbid AUD/PTSD. Additionally, individuals with comorbid AUD/PTSD displayed more severe drinking outcomes than individuals with only AUD and those without either disorder. This suggest that a dysregulated stress response system may underlie persistent negative affect and alcohol‐related behaviours in this population. Furthermore, while greater childhood trauma severity was associated with poorer NE outcomes, it did not amplify the difference between AUD and AUD/PTSD groups. This suggests that while childhood trauma is a critical risk factor for AUD/PTSD development, its effects on NE‐related dysregulation may be more generalized than specific to comorbid individuals.

### 4.1 Psychological NE Phenotypes

Consistent with prior research demonstrating dysregulation in NE in individuals with comorbid AUD/PTSD [2, 30, 31], depression and anxiety levels were higher among comorbid individuals than individuals with AUD only in our sample. To our knowledge, this was the first study to evaluate perceived stress in a comorbid nonveteran population. We found that perceived stress was higher in the comorbid group compared to the AUD‐only group. A previous study using U.S. veterans found that higher perceived stress was associated with a greater number of PTSD and AUD symptoms [32]. Current pharmacological interventions are not effective at reducing negative affect in individuals with comorbid AUD/PTSD despite improvement in AUD/PTSD symptoms [8]. Therefore, interventions that target negative affect, such as the Concurrent Treatment for PTSD and Substance Use Disorder Using Prolonged Exposure (COPE) protocol [33], may be well‐suited for this population.

Greater perceived stress and anxiety were associated with greater childhood trauma severity, consistent with prior evidence [12, 34, 35]. In the present study, depression was not significantly associated with childhood trauma severity, contrasting prior evidence [12, 35]. This suggests that dysregulation in anxiety and perceived stress may be more sensitive to childhood trauma severity than depression.

### 4.2 Physiological and Biological NE Phenotypes

Dysregulation within the NE domain also extended to biological systems. To our knowledge, no prior studies have examined biological markers of NE in individuals with comorbid AUD/PTSD, though many studies have examined these biological markers separately in AUD and PTSD groups. Previous studies reported elevated physiological NE phenotypes (i.e., HR and BP) among those with PTSD [36, 37]. In those with civilian and combat‐related trauma, heart rate and diastolic and systolic blood pressure were elevated [38] or similar [39] between those with and without PTSD. Further, alcohol dependence and alcohol consumption are positively associated with blood pressure [40]. In AUD individuals, resting heart rate is elevated [41] compared to non‐AUD individuals. Consistent with these findings, our results revealed that individuals with comorbid AUD/PTSD exhibited higher resting heart rates than those with AUD only, and both the AUD and comorbid groups exhibited higher resting heart rates and diastolic blood pressure than those without either disorder.

Previous studies have reported dysregulated levels of NE biomarkers in individuals with AUD and PTSD. Albumin and creatinine levels are altered in individuals with AUD or heavy drinkers [42]. Individuals with PTSD also displayed lower albumin and creatinine [43]. These differences did not reach statistical significance in the present study after correcting for multiple comparisons. HDL cholesterol was elevated among the AUD groups in the current study. Alcohol consumption, dependence and abuse are associated with higher HDL cholesterol [44], however evidence shows decreased or unaltered HDL cholesterol among individuals with PTSD [45]. Additionally, we found no differences in Hgb‐A1C levels between diagnostic groups, consistent with previous literature [42]. Our results showed no differences in CRP levels between comorbid and AUD groups, replicating previous research [45]. Although childhood trauma is also a predictor of increased allostatic load [15], we did not detect differences in NE biomarkers as a function of childhood trauma severity. In summary, these results suggest that individuals with a comorbid diagnosis exhibited dysregulated sympathetic nervous system activity compared to those with AUD only.

### 4.3 Alcohol Use Outcomes

Individuals with comorbid AUD/PTSD exhibited more severe drinking behaviours, greater alcohol dependence, problematic alcohol use and obsessive‐compulsive drinking than AUD‐only individuals. Previous studies have similarly reported higher average daily drinks and problematic alcohol use in comorbid compared to AUD‐only individuals [28, 45]. Given the greater levels of stress, anxiety and depression exhibited by the comorbid group relative to AUD and HC groups, these individuals may be drinking to cope with their NE, highlighting the importance of disrupting the relationship between NE and alcohol use to prevent the perpetuation of the addiction cycle. Negative affect remains high in individuals with comorbid AUD/PTSD following pharmacological treatment despite reductions in AUD/PTSD symptoms [8], which may increase relapse risk. The COPE intervention reduces negative affect and alcohol craving in individuals with comorbid AUD/PTSD [46], which as proposed by Berenz et al. [47], may be a crucial link between negative affect and drinking behaviour. However, though COPE reduces alcohol craving and negative affect, heavy drinking days are unchanged [46]. Further research is needed to assess if COPE and similar interventions are effective at reducing other alcohol‐related symptoms (e.g., alcohol dependence and obsessive‐compulsive drinking) that were significantly higher in individuals with comorbid AUD/PTSD in the current study and that may interact with negative affect to perpetuate alcohol drinking behaviours.

### 4.4 Childhood Trauma Severity

Compared to individuals with either disorder alone, individuals with comorbid AUD/PTSD have greater childhood trauma load, are more likely to have experienced multiple and varied types of childhood trauma, and are less likely to achieve remission from their PTSD if they have childhood trauma [48, 49]. The current study extended previous findings by evaluating differences in severity level of childhood trauma between individuals with AUD and comorbid AUD/PTSD. The comorbid group was the most likely to endorse severe/extreme childhood trauma compared to AUD and HC groups. Further, the specific type of trauma also matters [50]. Individuals with comorbid AUD/PTSD in our sample were more likely to endorse severe/extreme childhood trauma across all trauma types than individuals with only AUD or without either disorder. These results suggest that efforts to prevent childhood adversity may be useful in lessening the risk of developing comorbid AUD/PTSD as well as related psychiatric comorbidities and symptoms.

Childhood trauma is associated with greater alcohol use and greater risk of AUD [12, 51]. Khoury et al. (2010) reported that alcohol use was unaffected by PTSD symptom severity in individuals with a history of childhood trauma/maltreatment. However, the comorbid group in the present study exhibited more severe drinking behaviours than the AUD group regardless of the severity level of childhood trauma. These results may indicate that the severity of drinking behaviours may not be driven by childhood trauma alone.

## 5 Strengths and Limitations

Strengths include a large, diverse sample across a wide range of drinking behaviours, the broad range of NE phenotypes assessed, including both psychological and biological markers, and measuring perceived stress in the comorbid AUD/PTSD condition in a civilian population. Limitations include not including a PTSD‐only group due to a very small number of individuals with only PTSD in our sample. Also, the cross‐sectional study design and the use of lifetime rather than current diagnosis prevented us from knowing if or when the two disorders overlapped. Our sample may not be representative because of selection bias due to individuals being selected for AUD but not PTSD. Finally, we did not assess additional comorbid diagnoses, like depressive disorders, which may have contributed to the group differences observed.

## 6 Future Directions

Despite evidence that AUD/PTSD are characterized by hyperarousal and hypersensitivity to stress, very few studies have examined HPA axis dysregulation in the comorbid condition. Further studies should explore neuroendocrine response to acute stress and brain activity in areas impacted by both AUD/PTSD in individuals with AUD with and without PTSD to further understand the biological underpinnings of NE dysregulation. Longitudinal studies would elucidate the directionality of effects between childhood trauma, diagnosis and NE phenotypes. Obtaining a larger representation of a PTSD only group would allow us to assess differences in NE between PTSD and comorbid AUD/PTSD groups. Finally, exploring the impact of the age of childhood trauma exposure may be an important factor to consider in assessing NE outcomes in individuals with comorbid AUD/PTSD due to its impact on the occurrence of this comorbidity [50].

## 7 Conclusions

Comorbid AUD/PTSD was associated with greater psychological and biological markers of negative emotionality than AUD without PTSD, the latter a novel finding suggesting that impairment in the negative emotionality domain extends to biological systems in the comorbid condition. Individuals with comorbid AUD/PTSD also endorsed more severe childhood trauma and alcohol use. Taken together, these results demonstrate the importance of individualizing and implementing treatments that address the heightened negative emotionality, childhood trauma and alcohol use that contribute to a more severe clinical profile in comorbid AUD/PTSD compared with AUD alone.

## Ethics Statement

The study was approved by the Institutional Review Board at the National Institutes of Health Clinical Center. All procedures performed in this study were in accordance with the ethical standards of the institutional and national research committee and with the 1964 Helsinki declaration and its later amendments.

## Consent

Informed consent was obtained from all individual participants included in the study. Participants were provided with the details of the study, and their consent was documented prior to their participation.

## Conflicts of Interest

The authors declare no conflicts of interest.

## Supporting information


**Table S1** Estimated means of outcomes and group differences by diagnosis.

## Data Availability

The data that support the findings of this study are available from the corresponding author upon reasonable request. Permission to reproduce material from other sources has been obtained and is available upon request.
