# Supplementary material for: Markers of Negative Emotionality in Individuals With Comorbid Alcohol Use Disorder and Post‐Traumatic Stress Disorder: Role of Childhood Trauma
Source: Addict Biol. 2025 Apr 18;30(4):e70037. doi: 10.1111/adb.70037 (PMC12007917; doi:10.1111/adb.70037)
Supplement: Supplementary file 1 — Table S1 Estimated means of outcomes and group differences by diagnosis. [file ADB-30-e70037-s001.docx]

Supplemental Table S1. Estimated means of outcomes and group differences by diagnosis.

| Outcomes, mean (±SE) | **HC**  **n=502 (38.9%)** | **AUD**  **n=610 (47.2%)** | **CMB**  **n=180 (13.9%)** | **HC vs. AUD (p)** | **HC vs. CMB (p)** | **AUD vs. CMB (p)** |
| --- | --- | --- | --- | --- | --- | --- |
| PSS score | 11.84 (0.84) | 18.87 (0.48) | 21.10 (0.56) | **p<0.001** | **p<0.001** | **p=0.001** |
| MADRS score | 2.79 (0.85) | 9.28 (0.49) | 14.54 (0.56) | **p<0.001** | **p<0.001** | **p<0.001** |
| STAIT score | 30.93 (1.22) | 43.65 (0.90) | 48.52 (0.81) | **p<0.001** | **p<0.001** | **p<0.001** |
| Resting heart rate (beats per minute) | 62.06  (1.53) | 69.87  (0.87) | 70.20  (1.01) | **p<0.001** | **p<0.001** | p=0.793 |
| Diastolic blood pressure (mmHg) | 73.92  (1.31) | 78.67  (0.75) | 78.85  (0.87) | **p=0.002** | **p=0.002** | p=0.871 |
| Systolic blood pressure (mmHg) | 121.76  (1.79) | 126.42  (1.02) | 126.22  (1.18) | p=0.057 | p=0.057 | p=0.896 |
| Albumin (g/dL) | 4.39 (0.04) | 4.43 (0.02) | 4.42 (0.02) | p=0.667 | p=0.667 | p=0.667 |
| Creatinine (mg/dL) | 0.82 (0.02) | 0.80 (0.01) | 0.80 (0.01) | p=0.588 | p=0.588 | p=0.705 |
| Total cholesterol (mg/dL) | 179.30  (4.40) | 184.84  (2.51) | 178.19  (2.91) | p=0.408 | p=0.833 | p=0.219 |
| HDL cholesterol (mg/dL) | 60.75  (2.67) | 70.61  (1.52) | 66.19  (1.77) | **p=0.003** | p=0.090 | p=0.075 |
| HgbA1C (mmol/mol) | 5.35 (0.07) | 5.35 (0.04) | 5.32 (0.04) | p=0.990 | p=0.990 | p=0.990 |
| C-reactive protein (log mg/dL) | 0.181 (0.13) | 0.54  (0.08) | 0.51  (0.09) | p=0.064 | p=0.064 | p=0.816 |
| AUDIT total score | 4.19  (0.97) | 19.41 (0.55) | 23.15 (0.64) | **p<0.001** | **p<0.001** | **p<0.001** |
| OCDS total score | 3.28 (0.85) | 14.69 (0.49) | 17.71 (0.57) | **p<0.001** | **p<0.001** | **p<0.001** |
| ADS score | 2.52 (0.89) | 14.55 (0.51) | 18.02 (0.59) | **p<0.001** | **p<0.001** | **p<0.001** |
| Total lifetime drinks | 8834.55 (5341.40) | 39852.13 (3049.90) | 65575.14 (3539.98) | **p<0.001** | **p<0.001** | **p<0.001** |
| Age of first drink (years) | 17.33 (0.50) | 15.26 (0.29) | 14.47 (0.33) | **p=0.004** | **p<0.001** | p=0.061 |
| Heavy drinking days | 6.20  (3.04) | 43.68 (1.73) | 53.53 (2.02) | **p<0.001** | **p<0.001** | **p=0.001** |

Note. SE, standard error of the mean; HC, healthy control; AUD, Alcohol Use Disorder; CMB, comorbid AUD/PTSD. Age, sex, and race were included as covariates for all analyses. Clinical Institute Withdrawal Assessment for Alcohol Withdrawal (CIWA-Ar)) scores were included as a covariate for the psychological, physiological, and biomarker outcomes. Pairwise comparisons were adjusted for multiple comparisons using the Benjamini-Hochberg procedure.
